# Endospanin Is a Candidate for Regulating Leptin Sensitivity

**DOI:** 10.3389/fphys.2021.786299

**Published:** 2022-01-07

**Authors:** Richard L. Londraville, Matthew Tuttle, Qin Liu, Janna M. Andronowski

**Affiliations:** ^1^Program in Integrated Bioscience, Department of Biology, University of Akron, Akron, OH, United States; ^2^Division of BioMedical Sciences, Faculty of Medicine, Memorial University of Newfoundland, St. Johns, NL, Canada

**Keywords:** fish, bone, *LEPROT*, *OBRGRP*, leptin receptor (LEPR), leptin resistance

## Abstract

The hypothesis advanced is that endospanin, a highly conserved vesicle traffic protein in vertebrates, regulates leptin sensitivity in bone signaling. The effects of leptin on bones are well-studied but without consensus on whether the increases in leptin signaling stimulate bone gain or loss. The bone response may depend on leptin sensitivity, and endospanin is an established modulator of leptin sensitivity. An argument is advanced to develop zebrafish models for specific leptin signaling pathways. Zebrafish have well-developed molecular tools (e.g., CRISPR) and the advantage of non-destructive sampling of bones in the form of scales. Using these tools, experiments are described to substantiate the role of endospanin in zebrafish bone dynamics.

## Introduction

After the Friedman Lab’s original paper established leptin’s effects on appetite and metabolic rate (Zhang et al., 1994), thousands of studies expanded leptin’s pleiotropic effects to include sexual maturity, angiogenesis, immune/stress responses, and bone dynamics (reviewed in Copeland et al., 2011; Londraville et al., 2014, 2017; Guzmán et al., 2019; Münzberg et al., 2020). Leptin’s effects on bone metabolism in mammals is a central focus of intense research effort, leading to two conflicting models. One model predicts that leptin *reduces* bone mass through a hypothalamic relay and that peripheral leptin signaling does not play a significant role (Ducy et al., 2000). In contrast, another model asserts that leptin *builds* bone mass through signaling from both peripheral and central leptin pools (Turner et al., 2013). Although most studies use rodent models for studies that investigate leptin’s effects on bone, the disparate reported effects also extend to reports on humans (Table 1). Large-scale studies in humans demonstrate a *positive* correlation between leptin titer and bone mineral density (Ruhl and Everhart, 2002), although that correlation decreases in magnitude or turns *negative* after correcting for the body mass index (BMI) (Ruhl and Everhart, 2002; Meng et al., 2019). Whether augmented leptin signaling increases or decreases bone mass (or some correlate of bone mass, e.g., bone mineral density-a densitometric scan compared to a standard, or bone volume-density per measured volume of bone) remains unresolved.

**TABLE 1:** Effects of changes in leptin signaling on bones.

Study organism	Genotype/phenotype	Bone(s)	Treatment and result	Leptin signaling	Effect on bone	References
Rat	*fa/fa*	Femur	fa/fa rats compared to wt	↓	↓	Foldes et al., 1992
Mouse	*db/db*	Femur	*db/db* vs. wt after 8 weeks development	↓	↓	Takeshita et al., 1995
Mouse	OVX *ob/ob*	Tibia and vertebrae	ICV infusion of leptin in OVX *ob/ob*	↑	↓	Ducy et al., 2000
Rat	OVX wt	Tibia	Peripheral leptin implant in OVX wt	↑	↑	Burguera et al., 2001
Mouse	STZ-induced diabetic wt	Tibia	Peripheral leptin implant in STZ-diabetic wt	↑	←→	Motyl and McCabe, 2009
Rat	wt	Tibia	Hind limb unloading with peripheral leptin implant	↑	↑	Baek and Bloomfield, 2009
Mouse	*ob/ob*	Femur + vertebrae	Subcutaneous leptin injection	↑	↑	Turner et al., 2013
Rat	wt	Femur + vertebrae	Gene therapy to increase leptin expression in CNS	↑	←→	Turner et al., 2015
Human		Hip	Correlation between serum leptin and bone mineral density	↑	↓♂ ←→♁	Ruhl and Everhart, 2002
Human	Hypothalamic amenorrhea	Total skeleton	Subcutaneous leptin injection	↑	←→ (bone density) ↑ (alkaline phosphatase)	Welt et al., 2004
Human	Gastric bypass	Radius Hip Lumbar spine	Pre vs. post gastric bypass	↓	↓	Johnson et al., 2005
Human	Hypothalamic amenorrhea	Spine, upper and lower limbs	Intense exercise (ballet dancer)	↓	↓	Kaufman et al., 2002
Zebrafish	Morpholino knockdown of leptin	Otolith	knockdown vs. control	↓	↓	Liu et al., 2012
Zebrafish	High-fat diet	Scale	High-fat diet vs. control	↑	↓	Carnovali et al., 2018

*The table is a generalized overview tabulating the diversity of reported responses of bone to leptin. For each study, how the variable(s) changed leptin signaling (increase ↑, decrease ↓), and the reported response of bones (increase ↑, decrease ↓, or no change ←→) is listed. For example, fa/fa rats do not express functional leptin receptors; thus, leptin signaling is reduced compared with wild-type controls, and the reported response is a decrease in bone mass (Foldes et al., 1992). This table is a greatly simplified representation of the reported results; many more variables were tested in the studies than are represented. Similarly, how each study measured the response of bone is diverse (including bone volume, bone mineral density, bone area, enzyme activity, etc.) and is not intended to be equivalent across studies. fa/fa, leptin-receptor deficient rat; db/db, leptin-receptor deficient mouse; ob/ob, leptin-deficient mouse; wt, wild-type; OVX, ovaries removed; STZ, streptozotocin antibiotic (used to induce diabetes).*

Numerous studies provide important details that underscore the complexity of leptin’s action on bones. Initially, the discrepancy among models was attributed to administering leptin centrally (*via* the central nervous system, behind the blood-brain barrier) vs. peripherally (*via* vasculature). Central leptin delivery was reported to reduce bone mass (Ducy et al., 2000), whereas peripheral leptin administration increased bone density (Burguera et al., 2001), advancing the hypothesis that the total skeletal bone density was a balance of these positive and negative effects (Burguera et al., 2001). Turner et al. (2013) challenged this with a single study using various approaches to rescue leptin signaling in *ob/ob* mice (mutant mice that do not express circulating leptin); the results of those multiple approaches supported a bone-building role for leptin, primarily from peripheral leptin. Briefly, the *ob/ob* mice were given subcutaneous leptin injections (peripheral), or leptin gene therapy delivered into the third ventricle of the brain (central). Leptin injections increased the bone formation rate in *ob/ob* mice to that of (or above) wild-type, and the leptin gene therapy in the hypothalamus returned mineralized bone area in *ob/ob* mice to that of wild-type (Turner et al., 2013). Peripheral leptin’s effects are qualitatively different than leptin delivered through the central nervous system; central gene therapy that causes rats to lose weight does not change their cancellous bone volume when compared with control vectors (Turner et al., 2015) and the bone-specific elimination of leptin signaling does not affect the whole-animal metabolic rate in mice (Yue et al., 2016). In addition, bones respond to peripheral changes in leptin titer at lower doses than those that affect energy metabolism (Philbrick et al., 2017). Finally, it does not appear that leptin’s effects on bones are secondary to other effects. For example, *ob/ob* mice are sexually immature, and leptin can rescue wild-type sexual maturation (Chehab et al., 1996). Since estrogen signaling also affects the bone and contributes to bone remodeling (Beekman et al., 2019), leptin’s effects on bone may interact with leptin’s effects on estrogen signaling. Mice without ovaries still respond to increases in leptin signaling (Ducy et al., 2000; Burguera et al., 2001; Table 1). Further, Turner et al. (2017) used an estrogen receptor antagonist in *ob/ob* mice and demonstrated that leptin still rescues bone length and volume in the absence of estrogen signaling.

Adding to the complexity of the bone’s response to leptin is the fact that leptin signaling changes do not have equivalent effects on all parts of the skeleton, which may contribute to the diversity of effects reported (Table 1). Rodent models remove leptin signaling through mutations that eliminate leptin expression (*ob/ob*) or leptin-receptor expression (*db/db* mice and *fa/fa* rats). These mutant models generally have increased cancellous bone volume in the lumbar vertebrae and decreased bone mineral content and length in long bones. Generally, these changes reverse with leptin administration (summarized in Reid et al., 2018).

Clearly, the effects of leptin on bone in mammals are complex but real. Individual studies are carefully executed, with well-reasoned (but contradictory) conclusions. Hamrick (2004) proposed a synthesis of these results, hypothesizing that leptin promotes satiety, sexual maturity, and growth (bone deposition) in concert during adolescence, and inhibits these physiological axes during food limitation. Further, he proposed that changes in leptin sensitivity contribute to the diversity of the bone responses reported (Hamrick, 2007). We extend that idea and propose a mechanism for leptin sensitivity change. We advance the hypothesis that multiple bone cell types and their progenitors differ in their leptin sensitivity depending on leptin receptor expression, which changes across different physiological states and biomechanical stimuli. For example, *ob/ob* mice are extremely leptin sensitive due to high leptin receptor cell-surface localization (Tortoriello et al., 2007), and therefore, bone density responds to increased leptin signaling. Similarly, different bones are composed of different cell populations. These populations likely vary in leptin receptor expression and physiological stimuli; thus, leptin response is not uniform across all bone tissues. Below we develop the hypothesis that leptin receptor expression, and thus, leptin sensitivity, is modulated through endospanin.

### Endospanin Is a Regulator of Leptin Receptor Cell-Surface Expression

Endospanin was first described as the product of an alternate start-site under control of the same promoter as the leptin receptor (Bailleul et al., 1997). Originally named the leptin receptor overlapping transcript (*LEPROT*), or obese receptor gene-related product (*OBRGRP*), it was renamed endospanin by Séron et al. (2011) for its late endosomal/Golgi localization and membrane-spanning topology. Two paralogs of endospanins are expressed in mammals, with endospanin-1 facilitating endocytosis and lysosome targeting of leptin receptor; thus endospanin-1 expression is negatively correlated with cell surface (active) leptin receptors and negatively correlated with leptin sensitivity (Roujeau et al., 2019). The *LEPROT* gene copy number and leptin receptor (*LEPR*) expression are also negatively correlated in humans (Jeon et al., 2010). Further, endospanin-1 knockdown mice (targeting hypothalamus) are highly leptin sensitive and resistant to diet-induced obesity (Couturier et al., 2007). Endospanin-2 affects the skeletal muscle aerobic capacity (Lancel et al., 2018), and therefore also may be correlated with leptin sensitivity.

The endospanin-1 gene (*LEPROT*) is extremely conserved throughout the metazoa, including recognizable endospanin homologs in invertebrates, fungi, and plants (Londraville et al., 2017). Its *in silico* predicted structure contains several conserved amino acids, 4 transmembrane domains, and a highly conserved hydrophobic core (Londraville et al., 2017). Endospanins (as a family) may have a generalized vesicle traffic function. Indeed, endospanins are associated with RAS associated binding (RAB) proteins (small RAS GTPases) that facilitate vesicle traffic (Hirvonen et al., 2013). However, there is also evidence that endospanins specifically evolved to regulate leptin receptor expression. All classes of vertebrates (except one) have an endospanin gene with loci either within the *LEPR* gene (*via* an alternate start site as in humans) or within 150,000 bp of the leptin receptor gene and with no intervening gene (Londraville et al., 2017). Since genes in close proximity have similar transcription rates (Akhtar et al., 2013), it is a reasonable hypothesis that the transcription of endospanin and leptin receptors is co-regulated. The one exception to this *LEPROT/LEPR* gene synteny is in Teleost fish, where endospanin (*LEPROT*) and *LEPR* are on separate chromosomes (Londraville et al., 2017).

Our laboratory is testing the hypothesis that the interaction of endospanin and leptin receptor expression is fundamentally different in a Teleost (zebrafish). Adding zebrafish to the rodent models in the effort to understand the dynamics of leptin/bone signaling serves (at least) four purposes. First, repeating (in zebrafish) the approaches taken in rodents will add weight to either the “increase” or “decrease” camp (Table 1), assuming that the response to leptin is evolutionary conserved from fish to rodents. This is similar to the approach used to test the conservation of other leptin functions, such as its lipostatic or anorectic effects (Copeland et al., 2011; Londraville et al., 2014, 2017). Second, zebrafish have the advantage of non-destructive sampling of bones (scales), allowing for longitudinal studies within individuals. Third, zebrafish can regenerate bones (caudal amputation, reviewed in Tonelli et al., 2020). Finally, we can test whether gene architecture affects leptin signaling (e.g., proximity of endospanin and leptin receptor in the genome) as these differ between rodents and zebrafish. The fact that endospanin and leptin receptors have loci on different chromosomes in zebrafish allows selective knockout of each gene without the confounding concern that knockout of one will affect transcription of the other (as in mammals due to gene proximity).

## Discussion

### Testable Hypotheses on Endospanin Function in Zebrafish

Our overarching hypothesis is that endospanin modulates how bone responds to leptin signaling in zebrafish. Zebrafish have similar bone development to mammals, including the expression of *lepr* in hypertrophic chondrocytes, analogous to mice (Giovannone et al., 2019). Zebrafish also have mineralized scales, which can be non-destructively sampled, treated in cell culture, and serve as a proxy for the response of a skeletal bone (Carnovali et al., 2018). Zebrafish are already established as model organisms for several human skeletal diseases (Seth et al., 2013), including osteoporosis and osteoarthritis (Lleras-Forero et al., 2020). Zebrafish bones also respond to changes in leptin signaling (Liu et al., 2012; Carnovali et al., 2018; Giovannone et al., 2019). Recently, leptin-signaling knockout lines have been developed in zebrafish, similar to the *ob/ob* mouse (Michel et al., 2016; He et al., 2021; Kamstra et al., 2021).

**FIGURE 1:**
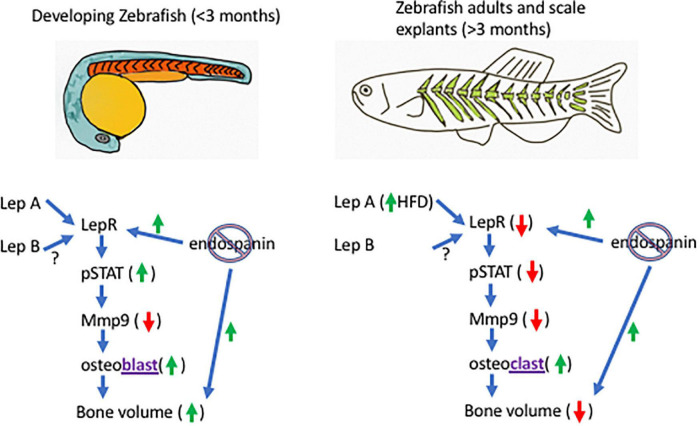
Model of the endospanin modulation of leptin sensitivity in zebrafish. In developing zebrafish (left), endospanin knockout is predicted to increase bone volume through increased cell-surface leptin receptor expression (LepR), increase leptin signaling [through increased phosphorylation of signal transducer and activator of transcription (STAT)], decrease expression of matrix metalloproteinase 9 (Mmp9) and increase the activity of osteoblasts. In adult zebrafish (right), high fat diet is predicted to decrease bone volume through lower leptin sensitivity (decreased cell-surface leptin receptor expression), lower expression of Mmp9, and increased osteoclast activity. Endospanin knockout in adults is predicted to restore leptin sensitivity (increased LepR surface expression) and bone volume.

In Teleost fish, as in mammals, some data support a positive correlation between leptin signaling and bone volume (Yang et al., 2008; Liu et al., 2012), and a negative correlation between leptin signaling and bone volume (Carnovali et al., 2018; Giovannone et al., 2019; Table 1). We suggest that this apparent conundrum can be resolved by understanding the endospanin function. In developing fish, robust leptin signaling [Lep binding to LepR, stimulating Jak/Stat signaling, increasing phosphorylation of signal transducer and activator of transcription (STAT), Figure 1] is hypothesized to result in osteoblast stimulation and increased bone volume (Yang et al., 2008; Liu et al., 2012). In adult fish, a high-fat diet (HFD) increases the expression of leptin (Carnovali et al., 2018). We hypothesize that this decreases the expression of LepR on the cell surface (leptin resistance), subsequently decreasing the Jak/Stat signaling and decreasing the phosphorylation of STAT, which lowers the activity of matrix metalloproteinase 9 [required for bone remodeling (Giovannone et al., 2019)], increases the activity of osteoclasts (Carnovali et al., 2018), and decreases bone volume. Bone response to leptin signaling, therefore, is hypothesized to depend on developmental contexts and leptin sensitivity.

We hypothesize that leptin sensitivity is modulated by endospanin. If endospanin functions in zebrafish as it does in mice, eliminating its expression (CRISPR knockout) would increase leptin signaling in a developing fish (by increasing leptin sensitivity), and therefore accelerate bone deposition compared with the wild-type. For adult fish on HFD, endospanin knockout would rescue the leptin sensitivity [as it does in mice (Couturier et al., 2007)], and therefore, rescue the bone volume (Figure 1). Bone remodeling in adult mice requires leptin signaling, as >90% of the mesenchymal stromal cells (MSCs) capable of forming bones express the leptin receptor (Zhou et al., 2014; Yue et al., 2016). Zebrafish MSCs that form bones also express LepR, which signals through Jak/Stat pathways (Giovannone et al., 2019). Although endospanin expression has not been documented in MSCs, it is documented to interact with RAB GTPases (Hirvonen et al., 2013) which are active in MSCs’ exosomes that participate in bone fracture repair (Blanc and Vidal, 2018). Therefore, the components of both leptin signaling and leptin sensitivity are (potentially) both present in MSC.

We hypothesize that the normal development of bones in wild-type fish would result from high leptin sensitivity, *via* limiting endospanin expression. This may be achieved by generating endospanin^–/+^ heterozygotes, or full knockouts *via* clustered regularly interspaced short palindromic repeats-CRISPR-associated protein 9 (CRISPR-CAS9) (Talbot and Amacher, 2014). Further, we hypothesize that normal bone turnover in adults would result from relatively low leptin sensitivity (higher endospanin expression). Increasing the expression of a gene product is typically more difficult than knockout approaches in zebrafish, although knock-ins have been achieved (Wierson et al., 2020). We predict that we can increase endospanin expression with diet, and adults on HFD would have very low leptin sensitivity (highest endospanin expression) and lowest bone volume, reflected in resorbed scales (Carnovali et al., 2018) and lower bone mineral density in the skeleton. In summary, we hypothesize that high leptin sensitivity promotes osteogenesis, and low leptin sensitivity promotes bone resorption (both mediated by endospanin). This hypothesis is informed by data reported for *ob/ob* mice, which have high leptin sensitivity, and respond to rescued leptin signaling with osteogenesis (Turner et al., 2013, 2015, 2017). If the hypothesis is supported, it would indicate that endospanin signaling is conserved, and not altered by the lack of endospanin/leptin receptor gene proximity in Teleosts. If not supported, it may be that endospanin and leptin signaling are decoupled in Teleosts. This could be pursued further by studying endospanin function in non-Teleost fish clades (gar, Family Lepisosteidae) that have the gene synteny shared with all other vertebrates (Londraville et al., 2017).

## Conclusion

Here we develop the hypothesis that leptin sensitivity variation underlies the response of bone to leptin, and propose that leptin sensitivity is modulated by endospanin expression. We assert that studies in non-rodent models, such as zebrafish, will aid in unraveling the seemingly contradictory results in rodents (mostly conducted with leptin-signaling deficient mutants), because zebrafish have similar molecular toolsets to rodents, with the advantage of non-destructive sampling (using scales). We develop this hypothesis by outlining an experimental design in zebrafish to further develop the role of leptin signaling in bone dynamics. Finally, we understand that leptin sensitivity is best known for its role in obesity (Liu et al., 2018), and indeed, mice made extremely leptin sensitive *via* endospanin knockdown are resistant to obesity (Couturier et al., 2007). In fact, endospanin’s regulation of leptin signaling (in an energy-balance context) has been the focus of most endospanin functional studies (Bailleul et al., 1997; Jeon et al., 2010; Séron et al., 2011; Lancel et al., 2018). Therefore, understanding endospanin’s role in bone dynamics, especially given the uniqueness of gene loci for these genes in Teleost fish, will likely pay dividends toward understanding the role of leptin resistance in obesity in addition to its role in bone dynamics.

## Nomenclature

Gene, mutant, and protein names are written in the convention for each species.

## Data Availability Statement

The original contributions presented in the study are included in the article/supplementary material, further inquiries can be directed to the corresponding author.

## Author Contributions

RL drafted the manuscript, which was reviewed and edited by MT, QL, and JA. All authors contributed to the ideas presented in this manuscript.

## Conflict of Interest

The authors declare that the research was conducted in the absence of any commercial or financial relationships that could be construed as a potential conflict of interest.

## Publisher’s Note

All claims expressed in this article are solely those of the authors and do not necessarily represent those of their affiliated organizations, or those of the publisher, the editors and the reviewers. Any product that may be evaluated in this article, or claim that may be made by its manufacturer, is not guaranteed or endorsed by the publisher.
